# Determination of ^241^Am in Environmental Samples: A Review

**DOI:** 10.3390/molecules27144536

**Published:** 2022-07-15

**Authors:** Haitao Zhang, Xiaolin Hou, Jixin Qiao, Jianfeng Lin

**Affiliations:** 1 Northwest Institute of Nuclear Technology, Xi’an 710024, China; zhanghaitaonint@163.com (H.Z.); linjf06@foxmail.com (J.L.); 2 Department of Environmental and Resource Engineering, Technical University of Denmark, DTU Risø Campus, 4000 Roskilde, Denmark; xiho@dtu.dk

**Keywords:** ^241^Am, environmental sample, determination, tracer application

## Abstract

The determination of ^241^Am in the environment is of importance in monitoring its release and assessing its environmental impact and radiological risk. This paper aims to give an overview about the recent developments and the state-of-art analytical methods for ^241^Am determination in environmental samples. Thorough discussions are given in this paper covering a wide range of aspects, including sample pre-treatment and pre-concentration methods, chemical separation techniques, source preparation, radiometric and mass spectrometric measurement techniques, speciation analyses, and tracer applications. The paper focuses on some hyphenated separation methods based on different chromatographic resins, which have been developed to achieve high analytical efficiency and sample throughput for the determination of ^241^Am. The performances of different radiometric and mass spectrometric measurement techniques for ^241^Am are evaluated and compared. Tracer applications of ^241^Am in the environment, including speciation analyses of ^241^Am, and applications in nuclear forensics are also discussed.

## 1. Introduction

The determination of americium-241 (^241^Am) in the environment is of importance in monitoring its release (both in controlled and accidental releases) and in assessing environmental impact and radiological risk. Several review papers have summarized the analytical methodologies for ^241^Am [1,2,3,4,5]. To the best of our knowledge, these works have made good reviews about the analytical methodologies of ^241^Am but have not specifically focused on ^241^Am determination in environmental samples. New radiochemical procedures and more sensitive measurement methods have been developed and applied for the determination of ^241^Am in environmental samples in recent years, but they have not yet been systemically reviewed. In addition, speciation analyses of ^241^Am in the environment and the environmental tracer applications of ^241^Am have not been well-addressed so far. This paper aims to critically review technical and methodological developments for the determination of ^241^Am in the environment, including the development of sample pre-treatment techniques, new radiochemical procedures and automated systems, and progress in measurement techniques, especially the capabilities of mass spectrometry. Apart from the above-mentioned aspects, the methodologies for ^241^Am speciation analyses in the environment and tracer applications of ^241^Am in geological and nuclear forensics studies are also discussed in this paper.

### 1.1. Nuclear Physical and Chemical Properties of ^241^Am

Americium, with an atomic number of 95, is one of the purely man-made elements. Americium has about 30 isotopes or isomers, with mass numbers ranging from 232 to 247 and half-lives ranging from 55 s to 7370 years. Among those isotopes, ^241^Am (*T*_1/2_ = 432.2 years) is the most important one and is formed in reactors as a product of the irradiation of plutonium with neutrons:

P239u(n,γ)P240u(n,γ)→P241u→14.2yearsβ−A241m

^241^Am is an alpha emitter with the energies of alpha particles (E_α_ = 5.388 MeV, 1.7%; E_α_ = 5.468 MeV, 85.2%; E_α_ 5.443 MeV, 12.8%) and gamma (E_γ_ = 59.6 keV, 35.9%) emission [6]. The gamma emission can be used to measure ^241^Am directly from some samples, such as highly contaminated soil. Most typically, ^241^Am is measured using alpha spectrometry. In the last few years, alpha spectrometry has been increasingly replaced by the more precise, accurate, and sensitive mass spectrometry. However, due to the high self-absorption of alpha particles and the interferences in mass spectrometric measurement, radiochemical separation is needed to concentrate and separate Am from interfering elements.

Americium is known in formal valence states of 0, III, IV, V, and VI, but only state III is prevailing in ordinary redox conditions. Due to comparable electronic configurations (c.f. Am [Rn] 5f^7^7s^2^ and Eu [Xe] 4f^7^6s^2^) and ionic radii (Am^3+^: 98nm and Eu^3+^:94.7nm), americium has similar chemical properties with lanthanides. The closely similar behavior of Am(III) and lanthanides becomes the main obstacle in separating americium from lanthanides. The formation of complex ions in aqueous solutions with inorganic ions or organic compounds is an important property of Am. The strength of the complexes of Am(III) with inorganic ligands is as follows:

PO_4_^3−^ > CO_3_^2−^ > OH^−^ > SiO(OH)_3_ > HPO_4_^2−^ > F^−^ > SO_4_^2−^ > H_2_PO_4_^−^ > SCN^−^ > NO_3_^−^ > Cl^−^ > ClO_4_^−^.

In groundwaters that are rich in carbonate, carbonate complexes of Am are the prevailing species in the pH range from 6–11. Americium is also strongly associated with organic matter, such as humic acid. The basis of the separation of americium and lanthanides on an anion-exchanger relies on the strong sorption of the anion complexes of Am(III) with SCN. Am(III) can be oxidized theoretically to Am(IV) because the standard electrode potential of the Am^4+^/Am^3+^couple in basic solutions is relatively low (0.4–0.5 V). However, the species of Am(IV) are unstable unless in the presence of a strong, complex agent, e.g., phosphates, and are reduced back to the trivalent state easily. To oxidize Am(III) to Am(VI), forming AmO_2_^2+^, a more powerful oxidizer such as (NH_4_)_2_S_2_O_4_ is needed [7]. The highly valent Am(V) and Am(VI) species are more stable in basic solutions compared to acidic solutions. Burns et al. [8] oxidized Am^3+^ to AmO_2_^+^ and stabilized the AmO_2_^+^ ions in acidic media with the presence of hypochlorite, which is possible to enable an effective group separation of lanthanides from actinides.

### 1.2. Sources of ^241^Am in the Environment

^241^Am is released into the environment through several different sources. First of all, as the result of atmospheric nuclear weapons testing from 1945–1980, controlled or accidental releases from nuclear reprocessing plants (Sellafield, La Hague, and Mayak), and nuclear accidents (Palomares in 1966, Thule in 1968, Chernobyl in 1986, and Fukushima in 2011), ^241^Am has been introduced directly to different extents into the environment [9]. Secondly, the level of ^241^Am in the environment has been increased due to the decay of the pure beta emitter ^241^Pu (T½ = 14.2 year) from nuclear denotations, either authorized or accidental releases, and should continue to increase for the next several decades. It has been estimated that, during the period of 1945–1980, about 142 PBq of ^241^Pu [10] was released into the environment by atmospheric nuclear weapons testing. Considering that the total deposition of ^241^Pu was about 108 PBq for the northern hemisphere to a reference date of 1965, about 3.0 PBq ^241^Am would have been generated by 2006 due to the decay of ^241^Pu [10]. In the same situation, the amount of ^241^Am released from the Chernobyl reactor was estimated to increase to 33.6 times the initial activity until 2058 [11], and the activity of ^241^Am should reach its maximum value approximately in 2090 [12]. Table 1 summarizes the different sources of ^241^Am and ^241^Pu in the environment. Up to this date, the majority of ^241^Am in the environment has originated from atmospheric nuclear weapons testing. The amount of ^241^Am released by either reprocessing nuclear plants or nuclear accidents accounts for a small proportion of the total inventory in the environment. It should be noted that the peak release of ^241^Am from the nuclear reprocessing plant at Sellafield occurred from 1971–1975, with the highest discharge of 118 TBq in 1974. After the initial peak, the discharge of ^241^Am from Sellafield reduced to about 8 TBq y^−1^ from 1978–1981 and then to about 0.04 TBq y^−1^ from 2005–2009 [13]. The La Hague nuclear reprocessing plant in France also reached its peak discharge of ^241^Am in 1974. From 1995–1999, the discharges of ^241^Am and ^241^Pu from La Hague were 0.31 TBq and 21.9 TBq, respectively. However, the total discharge of ^241^Am from La Hague has not been well-documented [14]. The determination of ^241^Am is an important topic in relation to health, nuclear waste management from nuclear reactors, the recycling and final storage of radioactive waste, the control of illicit nuclear activities, etc. Methods for the determination of ^241^Am in a wide variety of environmental samples include nondestructive gamma spectrometry, alpha spectrometry, mass spectrometry, etc., after chemical separation.

### 1.3. Distribution and Transfer of ^241^Am in the Environment

The distribution characteristics of ^241^Am in the environment are strongly influenced by the source and migration behavior of ^241^Am in different environmental compartments, such as atmospheric, terrestrial, and aquatic environments. The levels of ^241^Am vary with location, sample type, and the transport pathways within and between different environmental compartments. Table 2 summarizes the distributions of ^241^Am in some specific samples from different locations.

^241^Am is one of the most toxic transuranic nuclides due to its long half-life, α-particle emission, and especially its tendency to deposit in several key tissues and organs, such as the skeleton and liver, if it enters the human body. ^241^Am in the environment can be transferred into the human body through drinking water and the food chain. The World Health Organization (WHO) suggested that the level of ^241^Am in drinking water should be below 1 Bq/L [37]. The maximum permissible quantity for ^241^Am in the human body was reported to be 11.1 kBq (8.77 × 10^−8^ g) [38].

Attention has been also paid to recent trends in the transfer of ^241^Am from the food chain to creatures, as well as from soil to plants. Baigazinov et al. [39] presented the transfer parameters of ^241^Am to the tissues of horses from contaminated soil and feed at the Semipalatinsk Test Site (STS). The observed maximum transfer factor for ^241^Am was (72 ± 22) × 10^−5^ day∙kg^−1^ fresh mass in the liver of a mare fed with leachate from contaminated soil and feed. A.S. Mamyrbayeva et al. [40] described the excretion dynamics of ^241^Am from the muscle, liver, and bone of broilers after a 30-day application of contaminated feed. The results showed that ^241^Am mainly metabolized in liver and bone, and the activity concentration of ^241^Am in muscle was much lower. B.M. Bolotov et al. [41] reported that the concentrations of ^241^Am in human hair collected from the Semipalatinsk area were lower than 0.05 Bq/kg. However, there is still a lack of data on the transfer factors of ^241^Am from the food chain to human beings. In contrast, a number of studies have been carried out to investigate the uptake of ^241^Am from the soil into plants. The experiments conducted by Sokolik et al. [42] showed that the soil-to-grass transfer factor of ^241^Am decreased in the order of soddy-podzolic sand < soddy-podzolic loamy sand < alluvial soddy loamy sand < peat-bog. Plant species with different physical and chemical properties usually differ in their transfer factors for ^241^Am due to the variability in their metabolic processes and biological factors, the distribution of roots in the soil, and rhizosphere properties. Table 3 presents the transfer factors of ^241^Am from soil to plants, with most values at very low levels.

## 2. Sample Pre-Treatment and Pre-Concentration

### 2.1. Sample Pre-Treatment

The purposes of sample pre-treatment are to decompose the sample to release the target radionuclides into a homogenous solution to thus facilitate the subsequent chemical separation procedure. For soil samples and sediments, grinding, sieving, homogenizing, drying, and ashing are necessary before sample decomposition. Ashing aims to remove organic matters contained in the sample since, otherwise, they interfere with the performance of the chemical separation procedure. Wang [45] pointed out that ashing temperatures for soil samples should be carefully selected to avoid the formation of refractory fractions. An XRD analysis of soil samples revealed that plagioclase-like silicate materials were formed after high-temperature ashing, and 450 °C was recommended as an ideal ashing temperature.

The methods for sample decomposition can be divided into two categories: acid digestion and alkaline fusion. For acid digestion, aqua-regia leaching is frequently used to release actinides from the matrix and has been widely adopted by a number of laboratories to accommodate large-size samples for ^241^Am determination. However, it has been shown that leaching is not appropriate for soil containing refractory fractions. In the case of refractory residues associated with silicate lattices in the soil and air filter [46], HF in combination with other acids (typically HNO_3_ and HClO_4_) in an open vessel is a good choice for total dissolution of the matrix to release the entire Am content from the soil sample. Careful control of the physical and chemical conditions during total dissolution with an HF–HNO_3_–HClO_4_ system is necessary to prevent the formation of insoluble fluorides, such as AlF_3_ and CaF_2_. The process requires that the sample acid mixture is not evaporated to dryness in the initial decomposition. Practically, the addition of sufficient boric acid can prevent the formation of any insoluble fluorides. It is more efficient to perform sample decomposition in a closed system than in an open system. High-pressure microwave digestion systems with closed pressure relief containers have shown advantages of more vigorous digestion of ^241^Am at elevated temperatures and pressures, which not only reduces analytical time and consumption of the reagent, but also improves the operational safety [47,48,49]. However, closed microwave digestion systems might not be favorable to treat large amounts of samples.

Alkali fusion is an extremely aggressive method performed by melting the sample with a mixture of fusion flux (e.g., hydroxides, peroxides, carbonate, hydrosulfates, pyrosulfates, or lithium borates) in a graphite, nickel, zirconium, or platinum crucible at atmospheric pressure [1,50,51,52]. Due to the high operational temperature in the fusion process, the efficiency of decomposition by alkaline fusion is much higher compared to acid digestion. After cooling, the well-mixed molten cake is dissolved with diluted HNO_3_ or HCl. Maxwell [50] demonstrated that sodium hydroxide fusion could provide a rapid treatment for the analysis of ^241^Am in large soil samples (100–200 g). The main benefit of alkali fusion is the limited use of acids and the absence of HF in the pre-treatment process.

In general, acid leaching is the simplest method to treat large amounts of solid samples. However, the ^241^Am contained in some specific samples, such as vitrified samples, might not be completely released into the solution by acid-leaching because ^241^Am intrudes the crystal lattices of minerals. Incomplete sample decomposition gives rise to the underestimation of results. Total acid dissolution in many cases can dissolve the refractory fractions of samples, but it is time-consuming with high consumption of the acids and limited sample throughput. Alkali fusion can offer the complete decomposition of samples, and it is the most effective and aggressive method for decomposing solid samples containing silicates and refractory fractions. However, the extraneous salts introduced by the fusion flux are sometimes troublesome for the following chemical separation and measurement.

### 2.2. Pre-Concentration

The objective of pre-concentration is to concentrate the sample to a smaller volume and remove most matrix interferences. Coprecipitation is a commonly used method for the pre-concentration of Am from large-volume water samples, as well as the solution obtained after the pre-treatment of solid samples. The most frequently used reagents for the coprecipitation of Am are listed in Table 4. Generally, more than 95% of ^241^Am can be scavenged by coprecipitation. Sometimes, a combination of different reagents is used for the coprecipitation of ^241^Am. For example, calcium oxalate coprecipitation was used after ferric hydroxide coprecipitation to eliminate the interference of iron in the subsequent chemical purification for Am [53].

The marriage of mesoporous ceramics with self-assembled monolayer chemistry has created a powerful new class of environmental sorbent materials called self-assembled monolayer on mesoporous supports (SAMMSs). SAMMS materials offer extremely large surface areas (up to 500m^2^ g^−1^) and functionalities that have been fine-tuned to selectively capture ^241^Am and other actinides [64,65]. Am distribution coefficients were reported to be as high as 240,000 and 460,000 for two types of SAMMSs (Gly-UR SAMMS and Ac-Phos SAMMS, respectively) [64]. Since SAMMSs are effective in highly complex matrices such as blood, plasma, and urine, Yantasee et al. [66] used SAMMSs with an isomer of hydroxypyridinone (3,4-HOPO) for the selective pre-concentration of ^241^Am from blood and plasma to improve the detection limits of the analytical instruments. One appealing nanotechnology that uses magnetic nanoparticles (MNPs) conjugated with actinide-specific chelators (MNP-) for separating actinides from spent nuclear fuel solution was developed [67]. It utilized coated MNPs to selectively adsorb actinides (Np, Am, and Cm) onto their surfaces, after which the loaded particles were collected using a magnetic field. MNP-Che is an appealing technique the for pre-concentration of Am in water or solutions obtained after the pre-treatment of solid samples. The removal percentage of Am(III) by MNPs- DTPA in an acidic solution is over 90% after 30 min of sorption time.

## 3. Chemical Separation and Purification Procedures

Chemical separation and purification procedures are usually designed to concentrate and purify target radionuclides, which is imperative for low-level environmental radio assays. For the purification of ^241^Am, a variety of chemical separation procedures have been applied, including solvent extraction, ion-exchange chromatography, extraction chromatography, and combinations of two or more of these methods.

### 3.1. Solvent Extraction

Solvent extraction is widely used to separate ^241^Am in the reprocessing of spent fuel and the treatment of radioactive waste. Many extraction reagents have been involved in the separation of Am, including TTA, PMBP, TOPO, TOA, CMPO, HDEHP, and DDCP. For instance, a PMPB-TOPO extraction method was reported to purify americium in mosses and lichens [68]. Solvent extraction with PMBP in cyclohexane was used to purify americium from rare-earth elements [36]. Popov et al. [24] reported the use of 10% tri-iso-octylamine (TIOA) in xylene to separate americium from uranium, polonium, and plutonium in Bulgarian soil. One of the extractants, 2-hydroxy-2-trifluoromethyloctanoic acid (Hhfo), was synthesized and characterized for separating americium and lanthanides, and the maximum separation factor of Eu and Am by Hhfo reached 2.31 [69]. Due to the similar behaviors of Am(III) and lanthanides, it is always important to thoroughly separate americium from lanthanides to eliminate the interference of lanthanides in the measurement of Am. Three major groups of trivalent Am(III) ligands (O-donating, S-donating, and N-donating) have been proposed to separate Am from lanthanides based on the fact that americium forms slightly stronger complexes with ligands containing soft donor atoms than lanthanides. CMPOs are well-known O-donating ligands for separating Am(III). However, this type of ligand usually lacks discrimination between the same oxidation states of Am(III) and Eu(III), which results in a relatively low separation factor. Cyanex 301 is an S-donating ligand, and this sulfur-containing compound is a good example of an Am(III) chelator having a very high Eu and Am separation factor due to the preferable covalent binding of Am(III) to the relatively softer sulfur donor atom. N-donor ligands are classified as intermediates between O-donor and S-donor ligands with respect to extraction efficiency and Am(III) selectivity. A breakthrough came when Kolarik et al. synthesized the first BTP and found that it had remarkable extraction capabilities for actinides over lanthanides when contacted with high acidity in liquid–liquid extraction [70]. Panak et al. [71] investigated BTPs and BTBP, which were assumed to be promising solvents for separating Am(III) from lanthanides(III). The earliest BTPs had alkyl chains or alkyl-branched chains at the R position, such as *n*-Bu-BTP, isobutyl-BTP, and *n*-Pr-BTP. These extractants, along with other alkyl-substituted BTPs, displayed high selectivity for actinides over lanthanides in liquid–liquid extraction studies. Yuanlai et al. [72] synthesized a silica-based macroporous *iso*butyl-BTP/SiO_2_-P adsorbent by impregnating an *iso*butyl-BTP (2,6-di(5,6-diisobutyl-1,2,4-triazin-3-yl)pyridine) extractant into an acroporous SiO_2_-P support to directly separate trivalent Am from fission products (FPs) containing rare-earth (RE) groups in high-level radioactive liquid waste (HLLW). It was observed that the *iso*butyl-BTP/SiO_2_-P adsorbent exhibited good adsorption selectivity for ^241^Am over rare-earth (III) groups in a 0.01 M HNO_3_ solution and showed weak or no adsorption affinity to light and middle rare-earth (III) groups. The same group has also synthesized several other *R*-BTP/SiO_2_-P adsorbents (*R* = isohexyl, isoheptyl, and cyheptyl) and has investigated their fundamental properties, such as adsorption ability or stability [73,74,75]. Some other derivatives, such as CA-BTP [76,77], MOB-BTP [78], and CyMe_4_BTBP [79], have shown behavior more efficient for Am(III) extraction at lower concentrations. Nowadays, as great progress has been made in ion-exchange and extraction chromatography techniques, solvent extraction is no longer popular in the analysis of ^241^Am in the environment and is not suitable for batch-wise treatment due to its relatively high-complexity operations. However, solvent extraction still offers some attractive features when a single sample is required for analysis. For example, the separation of ^241^Am using solvent extraction with PMBP or TOA can be completed within a few hours.

### 3.2. Ion-Exchange Chromatography

Americium may be absorbed by either cation- or anion-exchange resin. The applicability of Chelex-100 cation-exchange resin was investigated for the separation of americium and samarium in aqueous solutions [80]. The maximum separation efficiency of Chelex-100 for trivalent lanthanides and actinides was achieved at pH 2.5. Increasing salinity (e.g., [Na^+^] and [Ca^2+^]), iron ([Fe^3+^]), and colloid concentrations in the solution resulted generally in decreasing the chemical recovery of Am. A procedure for the separation of Am from rare-earth elements was developed through step-by-step elution from KU-2 cation-exchange resin in the NH_4_^+^ form with α-hydroxyisobutyric acid (pH 4.75) [81]. Am^3+^ cations have a high distribution factor on cation resin at low acidities, which can be easily eluted from the cation exchangers by concentrated acids. However, insufficient selectivity was observed between Am(III) and lanthanides, which was due to the fact that Am^3+^ cations have a similar ionic radius and almost identical effective nuclear charges to Cm^3+^, Nd^3+^, and Sm^3+^. As a consequence, cation-exchange chromatography has not been widely applied for Am purification. Compared to cation exchange, anion exchange is a better choice for separating interfering alpha emitters from Am, especially relatively high levels of trivalent lanthanides. This is due to the fact that most of the matrix elements, especially lanthanides, are not able to form anion complexes under certain conditions, but anion complexes of Am(III) with SCN^−^ have strong sorption on anion exchangers.

### 3.3. Extraction Chromatography

Extraction chromatography (EC) is also called solid-phase extraction. Compared to solvent extraction, EC offers a number of advantages, including fast kinetics, high selectivity, and less reagent consumption and hazardous waste generation. Earlier studies have reported the application of supported HDEHP [82] and supported TOPO [83] for the separation of Am from Pu and U. Later, the supported HDEHP was developed and produced as a commercially available resin (LN resin) by Eichrom Co. A single column consisting of tri-n-octylaime (TONA) supported by microporous polyethylene was used to simultaneously separate ^241^Am, ^244^Cm, ^239+240,238^Pu, ^237^Np, and ^234,235,238^U. Because Am(III) was not retained on the TNOA extraction column, the effluent from the column loading was directly electroplated for Am measurement using alpha spectrometry [84]. Mohandas et al. [85] investigated the uptake of uranium and americium from nitric acid solutions with sulphonated phosphinic acid resin. The advantage of the sulphonated phosphinic acid resin, compared to phosphinic acid resin or conventional cation-exchange resin, was its greater capacity for the uptake of U(VI) and Am(III) from high-acid media. This advantage was maintained, even in the presence of NaNO_3_.

A series of EC materials have been developed for the separation of actinides by Horwitz and coworkers [86,87,88] at the Argonne National Laboratory during the 1990s, and later, Eichrom Co. made these materials commercially available. Commercial EC resins, including TRU, TEVA, UTEVA, DGA, DIPEX, and DIPHONIX, can facilitate efficient Am separation, and the characteristics of these resins are compiled in Table 5. Recently, several novel EC resins [79] were synthesized by the solvent impregnation of triazine ligands (CyMe_4_BTBP and CyMe_4_BTPhen) into Amberlite XAD7 and Amberchrom CG300 polymer supports. The Amberchrom-supported CyMe_4_BTBP resin achieved a weight distribution ration (D_Am_) of 170 within 60 min and a decontamination factor (DF) of >1000 for americium over lanthanides using column chromatography. The Amberchrom CyMe_4_BTPhen resin achieved a D_Am_ of 540 within 30 min and a DF for americium over lanthanides of 60–160.

### 3.4. Combined Procedures for ^241^Am Determination

**Figure 1:**
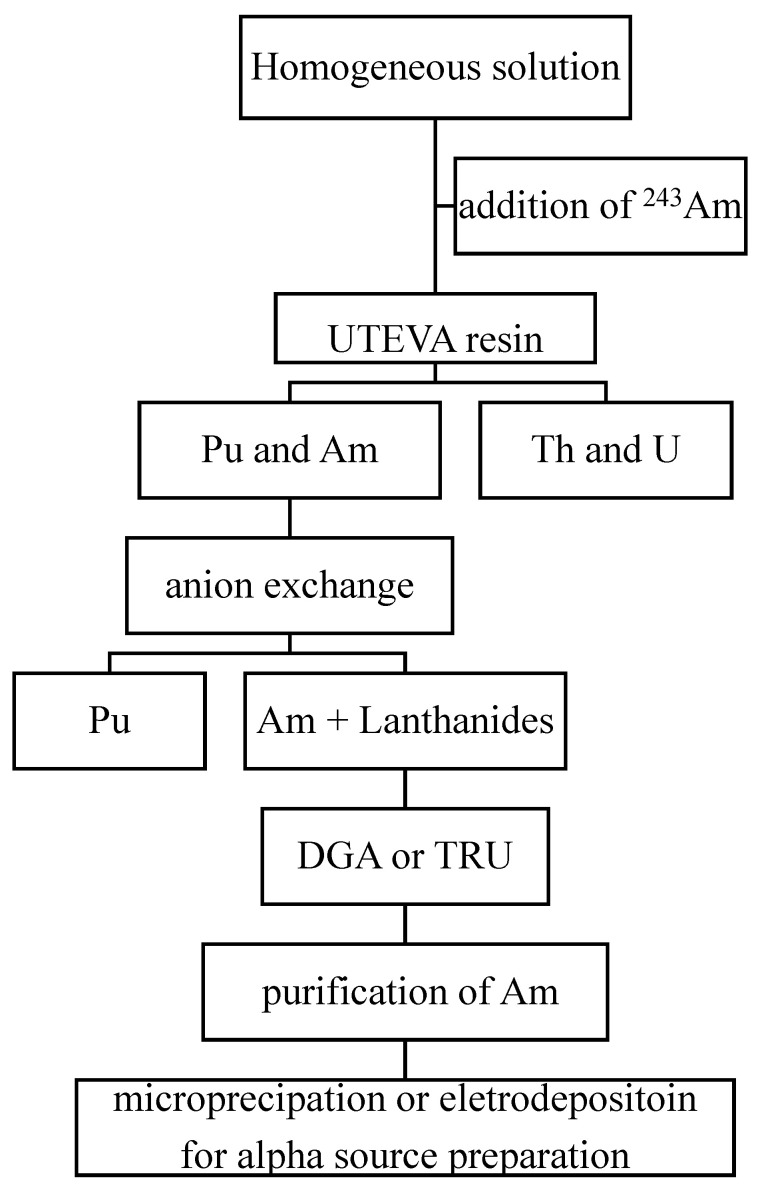
Scheme of a combined procedure for the determination of ^241^Am from an aqueous solution.

Although a single resin column, such as DGA, can provide a reasonably good separation of ^241^Am, it is impossible to meet the requirements of all cases for ^241^Am analysis in the environment. In recent years, many researchers have hyphenated different chromatographic resins to develop more effective procedures for ^241^Am determination. These methods sequentially separate ^241^Am and other actinides, providing advantages of reduced analytical time and cost. The principle of these combined procedures is based on the different absorption properties of different valent radionuclides on the EC resins. Figure 1 shows a typical scheme of a combined procedure for the determination of ^241^Am and other actinides in a homogeneous sample solution after pre-treatment and pre-concentration.

Table 6 summarizes the major characters of combined procedures for the determination of Am in environment samples that have been developed in recent years. Those combined procedures can meet the requirements for the determination ^241^Am in the environment.

### 3.5. Automated Systems for the Separation of Am

In the past decades, semi-automated and fully automated systems have been developed to analyze either single or multiple radionuclides in both emergency and routine operational situations [103,104,105]. These systems are suitable to separate not only Am, but also other radionuclides, such as Sr, Np, U, Th, and Pu isotopes. Most automated systems are based on dynamic flow approaches. The latest review [106] summarized systematically different flow approaches for automated radiochemical analyses in environmental, nuclear, and medical applications. Dynamic-flow-based approaches, including flow injection (FI), sequential injection (SI), multi-commuted flow injection (MCFI), multi-syringe flow injection (MSFI), multi-pumping flow system (MPFS), and pressurized injection (PI) techniques, have been developed and applied to meet analytical criteria under different situations. Recent testing has shown that flow techniques can be used for ^241^Am analysis in many situations, as summarized in Table 7. Specific automated systems for the determination of ^241^Am were developed using ion chromatography [107] and capillary extraction chromatography [108] separation prior to measurement.

## 4. Source Preparation

After chemical separation and prior to measurement, the purified Am fraction needs to be prepared in a certain geometric or chemical form to facilitate the subsequent quantitation using a radiometric or spectrometric method. Depending on the measurement method selected for ^241^Am, the criteria for the source preparation are different, but generally, fast turnover and high chemical yield are desired.

### 4.1. Source Preparation for Alpha Spectrometry

To obtain a thin, flat, and uniform alpha source is critical for achieving a high detection efficiency in alpha spectrometry measurement. Due to the short range of alpha radiation in matter, the thickness of the source should be limited to a few micrometers, otherwise the alpha spectra become degraded, and poor peak resolution makes it very difficult to evaluate the spectrum. Several methods have been used for ^241^Am alpha-source preparation, including evaporation, electrodeposition, micro-coprecipitation, and ion implantation. The pitfall of the evaporation method is that an alpha-source surface prepared by evaporation does not have strong adherence to a plate or disk. Mirashi et al. [115] compared electrodeposition with the drop deposition method and observed that the resolution of the alpha spectra obtained that were prepared using electrodeposition were better than those using the drop deposition method. The chemical yield of electrodeposition was strongly affected by pH, the concentration of buffer solution, the amount of impurities in the electrolyte, deposition time, temperature, etc. It was observed that, when Fe(III) concentrations >0.1 mM (~30 µg Fe) were found in NH_4_Cl solution (5 mL), only 6% of the ^241^Am could be electrodeposited [116]. A variety of electrolytes or buffers have been employed for ^241^Am electrodeposition, such as oxalic acid-NH_4_Cl solution [115], NaHSO_4_-Na_2_S_2_O_4_ buffer [117], H_2_SO_4_-(NH_4_)_2_SO_4_ buffer [53,84], Na_2_SO_4_ [118], NaHSO_4_-H_2_SO_4_-NH_4_OH buffer [119], NH_4_Cl [116], (NH_4_)_2_C_2_O_8_-hydroxyl ammonium sulfate-DTPA [120], and (NH_4_)_2_C_2_O_8_-(NH_4_)_2_SO_4_-DTPA [121]. Among these electrolytes, the NaHSO_4_-Na_2_S_2_O_4_ buffer solution is regarded as the most robust because the electrolyte can be pre-adjusted to an optimal pH, and no further adjustment is needed in the process. Jung-Suk et al. [122] evaluated the performances of NH_4_Cl, (NH_4_)_2_C_2_O_8_, and (NH_4_)_2_SO_4_ as electrolyte solutions for the preparation of americium sources for alpha spectrometry. The recovery of americium in the (NH_4_)_2_SO_4_ solution was found to be relatively low compared to those in the other solutions.

As an alternative to the electrodeposition method, the alpha source of americium can be prepared via micro-coprecipitation with rare-earth fluorides (often NdF_3_) [123] or hydroxide (such as cerous hydroxide, Fe(OH)_3_, and Sm hydroxide) [63,124,125]. Fluoride micro-coprecipitation methods have a higher selectivity for actinides and lanthanides than hydroxide methods. NdF_3_ micro-coprecipitation was reported to perform better than CeF_3_ and SmF_3_ [55] because NdF_3_ precipitated much more slowly and, thus, the precipitation was more homogeneous [123]. One of the key factors in the micro-coprecipitation process is that the sample size or the amount of rare-earth carrier must be limited so that the total mass of the precipitate does not exceed 100 µg to avoid undesirable degradation of the resultant alpha spectra. M. P. Dion [126] presented a novel method using ICP-MS, where the electron multiplier was removed, and a ‘‘collector’’ was fabricated to implant mass-selected ions for alpha spectrometry source preparation. This method produced thin, contaminant-free ^241^Am samples that yielded an energy resolution of 20 keV FWHM (full width at half maximum). Although electrodeposition is time-consuming and tedious (usually more than 1 h), alpha sources prepared with electrodeposition have much better resolutions and qualities than those prepared with micro-coprecipitation. The resolution of the α spectrum has been valued at about 20–30 keV for electrodeposited ^241^Am sources [117,119,120] but at about 40–50 keV for micro-coprecipitated sources [55,124]. Micro-coprecipitation procedures are much faster (within 30 min) and, generally, give much higher chemical yields (>98%) than electrodeposition (60–95%). Practically, electrodeposition is more favorable than micro-coprecipitation for americium alpha-source preparation to ensure the quality of the source.

### 4.2. Source Preparation for Mass Spectrometry

Among different mass spectrometric techniques, the most commonly used for ^241^Am measurement are ICP-MS, TIMS, and AMS. For ICP-MS measurement, the purified ^241^Am solution is preferentially prepared in weak HNO_3_ (around 0.5 mol L^−1^). This is usually achieved by evaporating the obtained americium fraction to dryness, destroying the organic matter, and re-dissolving with weak HNO_3_ solution. For TIMS measurement, the purified ^241^Am solution is typically reduced by evaporation to a very small volume (1 µL) that contains about 100–500 ng ^241^Am, which is loaded onto a high-purity filament (rhenium, tantalum, tungsten, etc.) and dried with electric heating. For AMS measurement, coprecipitation with NdF_3_ [91] or Fe(OH)_3_ [127] can be adapted for the source preparation. The precipitate is thereafter pressed into a target suited for AMS measurement. An optimized method using mixed titanium and iron hydroxide was developed, which showed promising results for the detection of femtogram levels of ^241^Am using AMS [128].

## 5. Alpha Spectrometry for ^241^Am Measurement

Alpha spectrometry is the most sensitive measuring technique to detect ^241^Am before high-sensitivity mass spectrometry is exploited. Alpha spectrometry can be performed using different types of detectors, such as gas ionization (Frisch grid) detectors, solid (e.g., ZnS(Ag)) and liquid scintillation detectors, magnetic spectrometers, nuclear track detectors, and semi-conduction detectors. A typical alpha spectrometer with an ion-implanted Si detector of 300–600 mm^2^ surface area and 100 µm thickness has a resolution of 17–25 keV in the energy range of 3–10 MeV, a counting efficiency of 15–30% for a source-to-detector height of 5–10 mm, and a background of 10^−4^ to 10^−5^ cps for a counting time of 10^5^ s. The FWHM of an alpha spectrum in the range of 20–60 keV depends not only on the performance of the detector but also on the quality of the source.

The complete separation of ^241^Am from the sample matrix is imperative for obtaining sufficiently thin alpha sources. It is of particular importance for samples containing relatively high levels of trivalent lanthanides and ^210^Pb from the americium fraction, which can interfere significantly with the measurement of ^241^Am by alpha spectrometry. Excessive lanthanides can degrade the alpha spectra, and ^210^Pb, through its grand-daughter ^210^Po (major alpha energy 5.30 MeV), interferes with the measurement of ^243^Am (major alpha energy 5.27 MeV), which is a yield tracer for americium separation. Since americium and curium behave chemically in very similar manners, the alpha spectra of ^241^Am often contain peaks of curium isotopes. The alpha peaks of ^241^Am,^244^Cm, and ^242^Cm are clearly distinguishable in alpha spectrometry so that the isotopes of ^242^Cm and ^244^Cm do not interfere with the determination of ^241^Am. In the case that there is no suitable isotopic tracer available for the determination of curium, it is reasonable that the yield of curium is calculated from the americium tracer. It is noteworthy that a slight deviation (<5%) might exist between americium and curium chemical yields in the same chemical procedure.

A suitable algorithm is very important to evaluate an alpha spectrum. A method based on the geometric progression decrease in the counts in the far tail of an alpha spectrum was developed for the simultaneous detection of plutonium, americium, and curium using alpha spectrometry [129]. For evaluating precision and accuracy, synthetic mixtures were prepared from solutions of enriched isotopes, and sources were prepared by direct evaporation and electrodeposition. Precision and accuracy of about 1% were demonstrated in the measurement of the ^241^Am/^239^Pu and ^241^Am/^233^U activity ratios using a silicon surface-barrier detector. Because of the relatively small energy difference, the peaks of ^241^Am (5.486 MeV, 85.2% abundance; 5.443 MeV, 12.8% abundance) and ^243^Am (5.277 MeV, 88% abundance; 5.486 MeV, 10.6% abundance) were partially overlapped. A series of synthetic mixtures covering a wide range (0.3 to 2.0) of ^241^Am/^243^Am alpha activity ratios from high-purity ^241^Am and ^243^Am solutions was employed to evaluate two algorithms used to account for the tail contribution due to energy degradation [130]. The results showed that precision and accuracy of about 1% could be achieved for ^241^Am/^243^Am activity ratios using alpha-spectrometry. In earlier years, special efforts have been made to resolve the overlapping of the ^238^Pu and ^241^Am peaks by means of an analytical function for fitting peaks in alpha spectra from Si detectors [131]. Similar to the early work of Bortels [131], M. P. Dion [126] presented the response of a silicon detector that was modeled as a convolution of a Gaussian model with one exponential function. These methods were based on the use of complex mathematical procedures to unfold the alpha spectra of radionuclides presented in the source. The impressive progress achieved by Devol et al. [132] was that ^238^Pu/^241^Am isotopic ratios of plated alpha sources were quantified by the alpha in a combination of conversion electron spectrometry using a cooled, passivated, ion-implanted planar silicon (PIPS) detector. However, the aforementioned methods have not found wide application for the measurement of ^238^Pu/^241^Am activity ratios due to their complexity. Up to now, none of the commercial spectrometers and spectrum evaluation software is available to distinguish the overlapping of ^238^Pu and ^241^Am peaks. Developing high-resolution alpha spectrometers should be regarded as an attractive avenue to perform the accurate determination of ^241^Am.

Alpha spectrometry has been used for many decades for ^241^Am measurement and is still a popular technique. For a typical Si detector assuming a 2-day measurement time, the limit of detection (LOD) for ^241^Am is obtained as 0.2–0.4 mBq/sample, which refers to 25–50 mBq/kg for 10 g of sample with an 80% chemical yield. The LOD can be improved by increasing the sample size, prolonging the counting time, and improving the chemical yield. The major disadvantage of alpha spectrometry is that it is time-consuming, especially when performing with low levels of ^241^Am, which can take from several days to several weeks depending on the concentrations of ^241^Am in samples. Attention should be paid in the purification of ^241^Am sources to avoiding any contamination from ^238^Pu, which emits alpha particles in similar energy range (5.499 MeV, 70.9% abundance; 5.456 MeV, 29.0% abundance) as ^241^Am. Due to these disadvantages, alpha spectrometry has been increasingly replaced by mass spectrometry in the last few years.

## 6. Mass Spectrometry for ^241^Am Measurement

More precise, accurate, and sensitive measurements of ^241^Am concentrations and isotope ratios at trace and ultra-trace levels are very necessary for environmental samples such as biological samples, soil, dust, and water. Mass spectrometric techniques are of interest due to their high sensitivity, multi-isotope capability, and high accuracy. Specially, ^241^Am/^243^Am isotope dilution mass spectrometry is the preferred method to determine the concentrations of ^241^Am in environmental samples precisely and accurately. Aggarwal et al. [5] reviewed mass spectrometric techniques for the analysis of americium several years before. In light of the significant progress in mass spectrometry techniques recently, the present status and trends of mass spectrometry for the measurement of ^241^Am are summarized in this review.

### 6.1. Inductively Coupled Plasma Mass Spectrometry (ICP-MS)

The powerful ICP-MS provides a fast and sensitive detection technique for long-lived radionuclides, such as actinides and ^99^Tc. In the initial phase of ICP-MS instrument development, the sensitivity of ICP-MS for the measurement of ^241^Am was not superior to alpha spectrometry [133]. Varga et al. [134] made an attempt to determine ^241^Am in Chernobyl soil using ICP-MS, but the limit of detection was only 104 fg/g. Nowadays, the analysis of ^241^Am (*T*_1/2_ = 432 years) can be carried out using ICP-MS with higher sensitivity and a lower detection limit [135]. ICP-MS has gained popularity compared to TIMS for ^241^Am determination in complex biological and environmental samples because of the less stringent requirements of sample purity, the ease of liquid sample introduction, the possibility to use another element as an internal standard for mass bias correction, the employment of an external calibration procedure, and the possibility for combination with an automated system. Chartier et al. [136] compared the performances of TIMS and ICP-MS to determine americium in spent nuclear fuels after separation with high-performance liquid chromatography. The results obtained with the double-spike isotope dilution method demonstrates that ID ICPMS was accurate and reliable for the determination of ^241^Am/^238^U and ^244^Cm/^238^U in spent reactor fuels. However, matrix effects, instrumental mass bias, spectroscopic and nonspectroscopic interferences, memory, and the carry-over effect still needed to be checked, minimized, and corrected. Potential interferences for the measurement of ^241^Am, including isobaric and polyatomic interferences, are listed in Table 8. These interferences could possibly be reduced by emphasis on the removal of interferences [137] using double-focusing sector field ICP-MS (SF-ICP-MS) [107] and high-resolution ICP-MS (HR-ICP-MS) [93] at the required mass resolution, as well as collision cells in quadrupole ICP-MS(Q-ICP-MS).

Zheng et al. [138] reported a rapid analytical method for determining ^241^Am in marine sediment using isotope dilution SF-ICP-MS combined with a high-efficiency sample introduction system (APEX-Q). A low limit of detection 0.041 mBq/g (0.32 fg/g) was achieved that was two times lower than the typical detection limit achievable by alpha spectrometry (ca. 0.1mBq). The phenomenon was observed that the isobaric interference with the determination of ^241^Am could be effectively removed when He-NH_3_ was used as a collision–reaction gas in Q-ICP-MS, while high sensitivity was still kept. Zhang and coworkers [26] developed a method for ^241^Am measurement using triple quadruple ICP-MS (ICP-MS/MS) with He-NH_3_ as a collision–reaction gas. The extremely low limit of detection of 0.091 fg/g was three times better than those using other types of ICP-MS methods. Very recently, the same group reported a novel method to determine ultra-trace levels of ^241^Am using ICP-MS/MS with O_2_/He-He as the collision–reaction gas. The polyatomic ions formed by the interfering elements (Pb, Hg, and Tl) could be completely eliminated, even when Cl^−^ was present in the solution. The detection limit of ^241^Am was as low as 0.017 fg/g [139]. Theoretically, the precision of isotope ratio measurements and the LOD can be improved by more than one order of magnitude using multiple-ion-collector ICP-MS (MC-ICP-MS) compared to single-collector ICP-MS. Steven J. Goldstein [140] applied MC-ICP-MS for the isotopic measurements of ^241^Am in environmental samples and obtained accurate results with a low detection limit of 1.4 fg. Table 9 compares the limits of detection (LODs) obtained with different types of ICP-MS instruments for ^241^Am measurement.

### 6.2. Thermal Ionization Mass Spectrometry (TIMS)

TIMS is a popular mass spectrometric technique for actinide isotope analysis to obtain isotope ratios with high accuracy (measurement trueness and precision). TIMS requires the element in pure chemical form to achieve high ionization efficiency. TIMS is free from polyatomic isobaric interferences and does not suffer from any memory or carry-over effect, as with ICP-MS. Because there is an inherent limitation of isotope fractionation in TIMS that leads to preferential evaporation of the lighter isotope, it is necessary to optimize the analysis conditions with a certified reference material (CRM) and to apply a mass fractionation correction factor to arrive at accurate isotope ratios. Thus far, no such CRM is available for americium. A meaningful attempt was made wherein three gravimetric mixtures with ^241^Am/^243^Am isotope ratios at, nominally, 1:1, 20:1, and 200:1 were prepared for calibrating TIMS instruments used for americium isotope measurement through an isotope dilution (ID) approach. The ID approach yielded analytical values with expanded uncertainties of ~0.1% (k = 2) [149].

An alternative method to overcome the limitation of isotope fractionation in TIMS is to employ total evaporation (TE) or ion current integration with a multi-collector detector system, which has been applied in U and Pu isotope analyses. The advantage of the TE methodology for isotope ratio measurement is that the sum-integrated ratio from the analytical technique is close to the true value. Alexandre Quemeta et al. [150] demonstrated that TIMS measurements with the TE method combined with isotope dilution could yield expanded uncertainties (k = 2) at 0.1% and 0.81% for the ^241^Am/^243^Am ratio and the concentration of ^241^Am. In a more recent study, they employed TIMS with total evaporation to measure Nd, Am, and Cm isotopes, and the uncertainty estimations were below 0.2% (k = 2) [151]. Multiple collector thermal ionization mass spectrometry (MC-TIMS) was evaluated for trace and ultra-trace levels of the isotope ratio analyses of actinides. The achieved high total efficiency and low background resulted in a detection limit of <0.1 fg ^241^Am using filament and cavity resin bead load techniques [152]. Up to now, the application of TIMS is still limited for the measurement of americium because this technique requires time-consuming and labor-intensive source preparation and cannot be hyphenated with online chemical procedures. Nevertheless, TIMS has the potential to meet high accuracy requirements when an americium isotopic CRM become available in the future.

### 6.3. Accelerator Mass Spectrometry (AMS)

Accelerator mass spectrometry (AMS) is presently one of the most sensitive analytical techniques for the determination of actinides. The reason for the high sensitivity of AMS is that the stripping process and acceleration of the ions to MeV energies provide both the destruction of the molecular isobaric background and a strong reduction in tailing interferences. The application of AMS to measure ^241^Am in the environment has become more and more popular in recent years. An attempt to use AMS to determine ^241^Am was performed by Kazi et al. [91] for soil samples, and the minimum detectable activity (MDA) of ^241^Am was achieved as 1.4 mBq (1.12 fg), much higher than the 0.3 mBq (0.24 fg) of MDA for alpha spectrometry. Quinto et al. [153] studied a method where actinides were concentrated from small amounts of groundwater and seawater via iron hydroxide coprecipitation and were directly pressed into sputter cathodes of AMS. The detection of the injected tracers for ^243^Am was nearly 8 × 10^−3^ fg/g. A method was tested to increase the beam current of americium for AMS using mixtures of PbF_2_ and NdF_3_, and the LOD of ^241^Am using this method was 1.8 fg [154]. Measurements of ^241^Am in oxide and fluoride coprecipitation matrices using AMS were also compared, and the results indicated that the fluoride anion beam method provided more than one order of magnitude better sensitivity than the oxide anion method. The detection limits of the fluoride anion method and oxide anion method were 0.3 fg and 1.5 fg, respectively [92].

Investigations on the performances and the potential backgrounds of americium analyses with low-energy AMS showed that the sub-fg range of ^241^Am could be determined relative to a ^243^Am tracer if the samples and AMS standards were prepared identically with regard to the matrix elements [155]. Xiongxin Dai [128] described a new bioassay method for the analysis of sub-fg levels of americium in large-volume urine samples using compact AMS, and the limit of detection for ^241^Am in urine was 0.1–0.2 fg/L. Another impressive work was the concentration of ^241^Am in groundwater from the Grimsel Test Site (Switzerland), with levels as low as 1.2 × 10^5^ atoms/mL (0.048 fg/mL) determined using AMS [127]. However, due to the high cost of an AMS facility, it is not suitable for routine measurements [156]. Complicated and expensive experimental equipment and time-consuming sample preparation still restrict the application of AMS in the measurement of ^241^Am.

### 6.4. Quality Control and Uncertainty for ^241^Am Determination

Since the radioactivity level of ^241^Am in most environmental samples is extremely low, the quality control in determining ^241^Am is increasingly important. Quality control over the accuracy of the data was assured by participating in comparison runs and by analyzing CRMs for ^241^Am [157]. CRMs represent important benchmarks in identifying methodologies, detecting training needs, upgrading the quality of laboratories’ performances, and assessing the validity of analytical methods. Polona Tavčar [6] reported the certified value of ^241^Am in some CRMs, including IAEA300 (sediment from the Baltic Sea), IAEA135 (marine sediment from the Irish Sea), soil-6 (soil from Austria), IAEA375 (soil collected after the Chernobyl accident), IAEA385 (Irish Sea sediment), IAEA368 (ocean sediment from Mururoa Atoll), and NIST-SRM 4350b (Columbia River sediment). Due to the decay of ^241^Pu, the certified value of ^241^Am presented in the CRMs should be corrected for in-growth from ^241^Pu.

To properly evaluate the uncertainty of concentrations of ^241^Am in environmental samples, the uncertainty components should be identified. Those components come from gravimetric links, measurement repeatability, ^241^Am decay and in-growth, background, and the accuracy and purity of trace ^243^Am. Zhang [26] suggested that the uncertainty of the ^241^Am concentrations in CRMs and other samples mainly came from the uncertainty of the atomic ratio of ^243^Am and ^241^Am measured with mass spectrometry (the same situation happened in measurements with alpha spectrometry). The uncertainty of the ^241^Am concentrations in environmental samples was generally less than 30%.

## 7. Speciation Analyses of ^241^Am in Environmental Samples

As the behavior of ^241^Am in the environment is strictly connected with its physico-chemical forms, the speciation of ^241^Am is very important to predict its transfer and to estimate its mobility and bioavailability. ^241^Am released to the environment can be present in different species, ranging from simple ions and complexes to colloids, particles, and fragments. The following information can be obtained via a speciation analysis of ^241^Am: (1) the confinement to particles of various sizes; (2) the distribution among various geochemical fractions (exchangeable, oxidizable, reducible, sulfide, etc.); (3) the distribution among the cation, anion, and molecular forms; and (4) the chemical characteristics of the radionuclide (its host compound, nearest ligand shell, degree of oxidation, etc.) [158].

### 7.1. Soluble Species of ^241^Am in Natural Water

In general, the trivalent state (Am(III)) is the only prevalent oxidation state in ocean- and groundwater [159]. However, under most environmental conditions, americium may exist as complex species in addition to Am^3+^. The soluble species of ^241^Am in natural water are summarized in Table 10 [160]. Hui et al. [161] modeled the speciation distribution and solubility of americium in Chinese Beishan groundwater using PHREEQC software. The results indicated that americium mainly occurred as [AmCO_3_]^−^ and [AmSiO(OH)_3_]^2+^ in neutral conditions, whereas AmOHCO_3_·0.5H_2_O and [Am(OH)_3_] became predominant in alkaline conditions. At the Australian legacy radioactive waste disposal site, the soluble species of americium in natural water were dominated primarily by cationic species, including Am^3+^, [AmCO_3_]^+^, [Am(OH)]^2+^, [Am(OH)_2_]^+^, and [Am(OH)_3_] [162]. The pH, as well as the concentration and types of ligands, in water affect the distribution of species compositions of ^241^Am.

### 7.2. Particle- and Colloid-Associated ^241^Am in Natural Water

According to the definition presented by Salbu et al. [163], particles are defined as entities with diameters larger than 0.45 μm, while colloids or pseudo-colloids are defined as localized heterogeneities ranging in size from 1 nm to 0.45 μm. The americium species is known to be readily stuck to particles and exist in colloid and pseudo-colloid forms. ^241^Am can form colloidal fractions in natural water fairly easily, especially in its lower oxidation states (Am(III)). This is due to the low solubility of some of its compounds and its tendency to hydrolyze even under relatively acidic conditions. Pseudo-colloids of ^241^Am in water are ionic species associated with colloids of other origins, such as organic fractions and mineral oxides (e.g., silica). The pH, HCO_3_^−^ content, metal concentration (aluminum), and presence of humic acids have all been identified as parameters influencing the formation of americium particulates. It was reported that americium was not readily able to be in particulate form (>0.45 μm) in most well- and streamwater in the Sarzhal region of the Semipalatinsk Nuclear Test Site [164]. Large proportions of americium (87%) were observed to be associated with mobile colloids in the submicron size range at the Australian legacy radioactive waste disposal site [162]. It was proved that, in acid, ion-poor water, only 17% of the ^241^Am was present as particles [165]. This implied that 87% of the ^241^Am existed in colloid, pseudo-colloid, and soluble forms. In more ion-rich water with neutral pH, a high amount of ^241^Am was found in particulate form, amounting to 67% in streamwater. Molero et al. [166] investigated the distribution of particulate americium in Spanish Mediterranean coastal waters by measuring concentrations of ^241^Am in suspended particulate matter after filtering (<0.22 μm) large volumes (200–300 L) of seawater samples. The results indicated that particulate americium constituted, on average, 45% of the total concentration in seawater, while soluble americium represented 55% of the total concentration. It is interesting to observe that the suspended particulate matter was enriched in ^241^Am by a factor of eight compared to ^239^Pu. This further confirmed the particle-reactive behavior of americium in natural water systems.

### 7.3. Fractionation Analyses of ^241^Am in Soil and Sediment

The most commonly used procedure for ^241^Am fractionation analysis in soil and sediment is sequential extraction. This is based on the method developed by Tessier [167] for the speciation of particulate trace metals in soil and sediment. This sequential extraction procedure can determine the fractionation of ^241^Am as several desired geochemical fractions that are leachable by reagents with different chemical compositions and strengths, such as exchangeability, binding to carbonates, binding to Fe-Mn oxides, and binding to organic and residual matter. Table 11 shows typical sequential extraction reagents and conditions used for the fractionation of ^241^Am.

The fractionation results of ^241^Am are very different with respect to different solid samples. In sea sediment, americium is always associated mainly with carbonates and organic matter, and the insoluble fractions are generally high. The distributions of ^241^Am fractions in sea sediments of different origins are presented in Table 12. In agricultural soil in the UK and western Europe, most of the ^241^Am was associated with organic matter [168]. In floodplain soil of the Yenisei River, americium was observed to be mostly associated with highly mobile organic matter, such as fulvo acids [159], while >70% of the ^241^Am was associated with organic matter in the Yenisei river sediment [169]. The distribution of ^241^Am in sediment traps from Lake Michigan showed that 75% of the ^241^Am was distributed in the Fe and Mn oxide fractions [170]. Americium was associated in Rocky Flats soil in the following order: sesquioxide (45%) > water soluble fraction (16%) > refractory silicate (14%) > carbonate (12%) > organic fraction (8%) > exchangeable fraction (6%) [171]. For samples from a former radioactivity laboratory during dismantling activities, most of the americium in solid residue was associated with carbonates (~18%), oxides (~41%), and residual phases (~32%). However, americium in tank mud samples demonstrated a more uniform distribution among carbonates (~29%) and organic (~36%) and residual (~24%) matter [172]. Lujaniena et al. [173] observed that ^241^Am was associated with acid solubles (41%) and residues (38.5%) in aerosol samples collected during the Chernobyl accident. As for contaminated soil samples from nuclear weapons test sites, a significant portion of the ^241^Am is associated with the residue phase. For example, Yanmei et al. [174] reported that 53–83.6% of americium remained in the residue phase in contaminated soil from western China. Similar results also occurred in contaminated soil from the Semipalatinsk Nuclear Test Site. These results indicated that americium released from nuclear weapons testing had limited mobility, and thus, its transfer and migration in the environment was not significant. Comparing the migration of ^241^Am and ^239,240^Pu in successive layers of Chinese forest, grassland, and desert soils, the migration behaviors of ^241^Am and ^239,240^Pu were rather similar; both velocities were less than 0.3cm/y in diverse types of soils [25].

## 8. Tracer Applications of ^241^Am in the Environment

### 8.1. ^241^Am as a Time-Marker for Sediment Dating

As an alternative to ^137^Cs, which has wide-spread applications for benchmarking sedimentation rate, applications of ^241^Am for sediment dating [177,178,179] or sedimentation rate in aquatic environments [180] have been reported occasionally. This method assumes that the ^241^Am level of fresh nuclear weapons test debris is essentially zero, and its presence in older deposits is through the in-growth of ^241^Pu in weapon-fallout-derived release. According to the reconstruction data of the cumulative ^241^Pu and ^241^Am inventories for the northern hemisphere since 1954, the distribution of ^241^Am was dominated by a peak in 1963, with ca. 20% of the inventory being attributed to that year [181]. In many cases, the value of ^137^Cs as a dating tool has been significantly reduced by the evident mobility of this isotope and its consequent limitation. However, ^241^Am is a preferable time-marker for a growing dataset from lakes, as well as regional and coastal seas, with a wide range of pH values, suggesting that ^241^Am is considerably less mobile than ^137^Cs and can provide a useful means of sediment-dating from the early 1960s, where the ^137^Cs signal has been significantly decayed. When the specific activity of ^241^Am in different layers of a sediment core are determined, it is reasonable to use the peak of ^241^Am activity to benchmark the year 1963 and to calculate the average accumulation rate. The half-life (432 years) of ^241^Am ensures that it remains detectable in lake sediment for several centuries, while ^137^Cs decays away during this period. The main challenge in applying ^241^Am as a time-marker lies in its low concentration. ^241^Am has not been widely adopted, partially as a result of difficulties in the rapid and reliable measurement of its trace levels.

### 8.2. ^241^Am Signatures in Nuclear Forensics

Nuclear forensics has been defined as the “analysis of intercepted illicit nuclear or radioactive material and any associated material to provide evidence for nuclear attribution”. This scientific analysis aims at providing clues about the intended use of the material and its history, providing investigative leads and possibly leading to a source attribution [182]. Many characteristic parameters (so-called signatures), including the ratio of ^241^Am with other isotopes (^241^Pu and ^239+240^Pu), often provide important information related to the source term and history of the materials.

**Figure 2:**
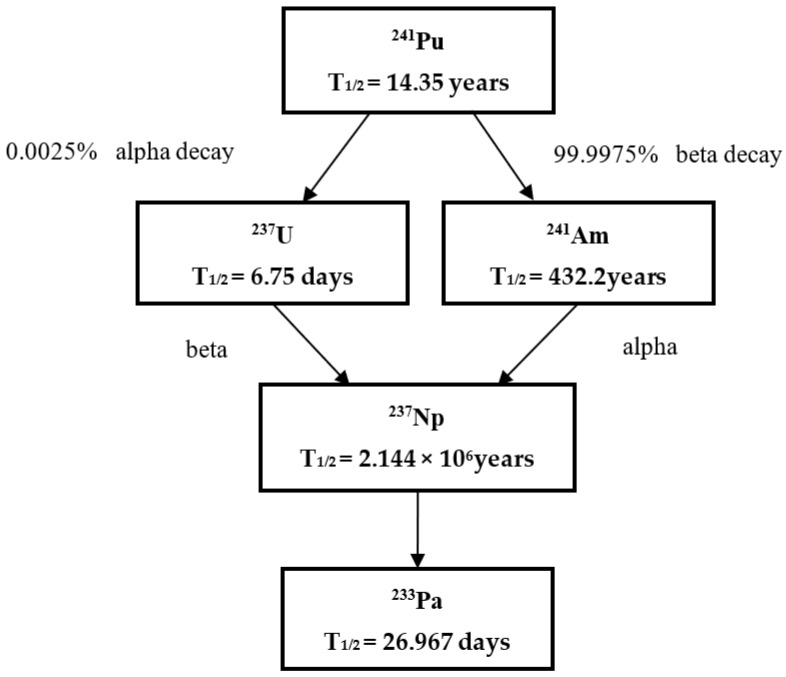
The decay chain of ^241^Pu.

The ‘age’ of plutonium material, as one of the key signatures which refers to the time elapsed since the last purification from its progeny, is the first parameter to be determined when deducing the history of plutonium material in nuclear forensics. The age of plutonium material can be determined in principle using four parent–daughter relations: ^238^Pu–^234^U, ^239^Pu–^235^U, ^240^Pu–^236^U, and ^241^Pu–^241^Am. Actually, the most important method for determining the age of plutonium is based on the ^241^Pu/^241^Am ratio due to the relatively short half-life of ^241^Pu (14.2 years). Therefore, the measurement of trace ^241^Am in plutonium material plays an important role in the age estimation of plutonium material. Figure 2 shows the decay chain of ^241^Pu.

Many methods have been developed for the dating of plutonium in recent years. The nondestructive method using gamma spectrometry is based on measuring the specific activities of ^241^Am and ^237^U, which approach equilibrium with ^241^Pu to obtain the ^241^Pu/^241^Am ratio [183]. This method needs at least 100 mg of plutonium material for the measurement and showed excellent results for samples with ages ranging from 8–20 years [184]. M. Wallenius [185] determined the ^241^Pu/^241^Am ratio using TIMS and described a methodology allowing for the accurate determination of several almost ’30-year-old’ plutonium materials. Zhang et al. [186] developed a method to determine the ^241^Pu/^241^Am ratio in plutonium material using alpha spectrometry and TIMS. This method attempted to avoid the use of an isotope dilution analysis so that it could be applicable for labs that could not obtain suitable isotope tracers, such as ^243^Am and ^242^Pu isotopes. Yan Chen [187] compared different detection methods, such as MC-ICP-MS, alpha spectrometry, and liquid scintillation counting (LSC), for age determination from the ^241^Pu/^241^Am ratios. The study showed that MC-ICP-MS provided the most accurate and precise ‘age’, with a typical precision of 1.5−3% for an ng amount of Pu material. However, the combination of alpha spectrometry with LSC produced 5−10% negatively biased results. The reason for this difference was attributed to the high uncertainty of the LSC measurement of ^241^Pu. Mats Eriksson [188] analyzed the americium signatures of five isolated, hot particles from the Thule accident (1968) using alpha spectrometry and ICP-MS. From the activity ratio of ^241^Pu/^241^Am, the ages of weapon-grade plutonium materials were estimated to be from the late 1950s to the early 1960s. This was in good agreement with [189], who estimated the time of production of the material to be 1960 ± 4.

All of the above-mentioned methods have relied on the hypothesis that there were no daughters (^241^Am, ^235^U, ^234^U, and ^236^U) existing in the plutonium material at the initial time. Zsolt Varga described a method for the preparation and validation of plutonium age-dating reference materials. The age values obtained for the test samples using the four different parent–daughter pairs (chronometers) were in excellent agreement and were also consistent with the known production date [190]. However, for US weapon-grade plutonium, ^241^Am/^241^Pu often gives significantly larger values for age than U isotopes such as ^235^U and ^236^U. The only reasonable explanation for this observation is that, when the uranium isotopes are removed from the plutonium sample for the last time, some americium is left in the material. In fact, it is still a challenge to identify freshly produced plutonium material through parent–daughter pairs. H.T. Zhang [186] reported a minimum reachable age and estimated that it was possible to distinguish only 134 days of newly produced plutonium materials from aged ones through the ^241^Pu/^241^Am ratio.

The inventory of ^241^Am and the isotopic ratios in environmental samples can provide clues to attribute the origins of ^241^Am and other isotopes. Several successful cases have been reported recently. Lichens and mosses from coastal zones of the Canadian Arctic and Alaska were sampled, and the analysis of the isotopic ratio exhibited the dominant contribution of the global fallout (SNAP 9A satellite re-entry fallout) for the presence of ^241^Am and plutonium isotopes [28]. Pierre-Andre Pittet [191] analyzed ^241^Am, ^241^Pu, ^239+240^Pu, and ^90^Sr in a few selected soil samples obtained near the nuclear reprocessing plant of La Hague. The results revealed the presence of previous environmental contamination originating from several incidents at the La Hague site involving atmospheric transfer and leaks in flooded waste pits. The radioactivity of ^241^Am and plutonium isotopes in soil cores from the Gambier Island (French Polynesia) were higher than the global fallout at this latitude, thus confirming that the dominant source of these radionuclides was from the local fallout during the 1970s of the French atmospheric tests of Moruroua and Fangataufa (located nearly 400 km from Gambier) [192].

## 9. Conclusions and Perspectives

As a result of atmospheric nuclear weapons testing and controlled or uncontrolled releases from nuclear reprocessing plants and nuclear accidents, ^241^Am has been introduced to different extents into the environment. The measurement of ^241^Am in the environment is of importance in monitoring its release and in assessing the environmental impact and radiological risk; thus, effective analytical techniques are needed. The separation of trivalent americium is always an issue in actinide chemistry due to lack high-efficiency procedures for americium and of high selectivity between americium and lanthanides. The most impressive progress might be the development and wide application of commercial extraction chromatographic resins, which continue to play an important role in the chemical separation of americium. Combined procedures based on different resins are aimed at finding more efficient and effective procedures for ^241^Am determination. Automated systems focus on increasing the analytical speed and throughput and reducing the lab intensity. Alpha spectrometry has been used for many decades for the measurement ^241^Am and continues be a popular technique due to its low cost. ICP-MS has shown higher sensitivity than alpha spectrometry and has begun to be applied more often for the determination of ^241^Am in the environment in recent years. More research works may be needed on the speciation of ^241^Am in soils and sediments, as ^241^Am is potentially a more soluble isotope than ^239^Pu. The tracer applications of ^241^Am in the environment have made remarkable progress. ^241^Am as a time-marker has been applied for sediment dating. The ratios of ^241^Am with other isotopes (^241^Pu and ^239+240^Pu) are important signatures, providing important information related to the history of materials and the origins of sources in nuclear forensics.

## Figures and Tables

**Table 1:** Sources of ^241^Am and ^241^Pu in the environment.

Source	The Activity of ^241^Am	The Activity of ^241^Pu	Release Period
Atmospheric nuclear weapons testing	13 PBq	142 PBq [10]	1945–1980
Reprocessing operations at Sellafield	542 TBq	22 PBq	1952–1992
	890 TBq [15]	-	up to 1990
Reprocessing operations at La Hague	-	12 PBq [14]	1967–1995
	310 GBq	21.8 TBq	1995–1999
Aircraft accident in Thule, 1968	0.20 TBq [16,17]	4.6 TBq [16,17]	2002
Aircraft accident in Palomares, 1966	0.1 TBq [18]	-	1966
Nuclear power plant accident in Chernobyl, 1986	0.99 MBq	6 PBq [10]	1986
Nuclear power plant accident in Fukushima, 2011	89 MBq [19]	14 GBq [19]	2013

1 PBq = 1 × 10^15^ Bq; 1 TBq = 1 × 10^12^ Bq; 1 GBq = 1 × 10^9^ Bq; and 1 MBq = 1 × 10^6^ Bq.

**Table 2:** Distributions of ^241^Am in some specific locations.

Location	Sample Type	Concentration of ^241^Am	Reference
France	cultivated soil (0–20 cm)	(45 ± 7) × 10^−3^ Bq/kg	[20]
Fukushima Dai-ichi NPP, Japan	surface soil (0–2 cm)	0.01–2.44 Bq/kg	[21]
	litter	0.012–1.64 Bq/kg	
Vilnius, Lithuania	aerosol	1–24.9 nBq/m^3^	[22]
New Mexico, USA	soil (0–2 cm) in the vicinity of the USA Waste Isolation Pilot Plant	0.003–0.067 Bq/kg	[23]
Bulgaria	surface soil (0–5 cm)	0.019–0.302 Bq/kg	[24]
China	forest, grassland, and desert soil cores	0.13–0.37 Bq/kg	[25]
Seven locations, China	soil (0–5 cm) collected from Hebei, Henan, Shandong, Inner Mongolia, Xinjiang, Sichuan, and Guangdong	0.041–0.221 Bq/kg	[26]
Prague, Czech	soils (0–5 cm) around nuclear research center	0.12 Bq/kg	[27]
Canadian Arctic and Alaskan tundra	lichens and mosses	0.50 Bq/kg	[28]
Italy	mosses	0.180–0.770 Bq/kg	[29]
	lichens	0.200–1.93 Bq/kg	
Peninsular Malaysia, east coast	surface seawater	0.5–1.9 mBq/m^3^	[30]
Mururoa and Fangataufa Atolls, French Polynesia	groundwater	≤0.008 Bq/L	[31]
Northwest Pacific Ocean	bottom sediments	0.44–10 Bq/kg	[32]
Aegean Turkish coast	marine sediment	0.003–0.33 Bq/kg	[33]
Black Sea coast	sediment	0.043–0.187 Bq/kg	[34]
Irish Sea	sediment	2.61–1894 Bq/kg	[35]
Ligurian Sea	sediment	0.09–0.14 Bq/kg	[36]

**Table 3:** Transfer factors for ^241^Am from soil to plants.

Species	Transfer Factors /m^2^·g^−1^	Remarks
Rice	2.5 × 10^−3^	In France [20]
Cereal grains	1.5 × 10^−7^ to 7.7 × 10^−1^	IAEA-recommended
Cowberry, stems and leaves	5 × 10^−4^	In Finland [43]
Billberry, stems and leaves	2 × 10^−4^	
Billberry, berries	9 × 10^−5^	
Lingonberry, stems and leaves	4 × 10^−4^	
Lingonberry, berries	1 × 10^−4^	
Elytrigiarepens	1.4 × 10^−7^	Contaminated regions in Belarus after Chernobyl accident [42]
Gramineae	1.0 × 10^−6^	
Carex	2.9 × 10^−6^	
Conium	6.0 × 10^−7^	
Rhinansus	3.4 × 10^−7^	
Moss	4.0 × 10^−6^	
Circiumarvens	1.9 × 10^−6^	
Poapratensis	1.0 × 10^−6^	
Leafy vegetables	3.6 × 10^−6–^3.5 × 10^−5^	[44]
Edible part of non-leafy vegetables	9.0 × 10^−5–^1.0 × 10^−4^	
Tubers	8.4 × 10^−6–^1.3 × 10^−5^	
Root crops	6.9 × 10^−6–^4.0 × 10^−5^	

**Table 4:** The most frequently used reagents for the coprecipitation of ^241^Am.

Reagent	Recovery/%	Function	Reference
Ferric hydroxide	>92	Pre-concentration	[54]
Ferrous hydroxides	>93	Pre-concentration	[7]
Lanthanide fluorides (NdF_3_ and CeF_3_)	>95	Pre-concentration, α source preparation	[50,55,56]
Calcium phosphate	>95	Pre-concentration	[50]
Bismuth phosphate	>95	Pre-concentration	[57]
Lanthanide hydroxide	>95	Pre-concentration, α source preparation	[58]
Manganese dioxide	>95	Pre-concentration	[54,59]
Mix of ferric hydroxides and barium sulfate	>95	Pre-concentration	[60,61]
Goethite (α-FeO(OH))	>95	Pre-concentration	[62]
Sm hydroxide	92.7	α source preparation	[63]

**Table 5:** The characteristics of different commercial extraction chromatographic resins.

Name	Extractant	Supporter	Characteristics	Application	Remarks	Literature
TRU	CMPO-TBP	Amberlite XAD-7	Am(III) retained on the resin; separated Am from tri, tetra, and hexavalent actinides	Determination of Th, U, Np, Pu, and Am(Cm) in sediment and swipe samples	Fe(III) retained on resin and competed with Am	[89]
TEVA	Aliquat 336	Amberchrom CG-7ms	An analogue to anion-exchange resin; retained tetravalent actinides, but Am(III) was only slightly retained from nitric or hydrochloric solutions; Am(III) was retained effectively as Am(SCN)_4_^−^	Separation of Am(III) from lanthanides	A good choice for separating Am(III) from lanthanides	[87]
UTEVA	dipentyl-pentyl phosphonate	Amberlite XAD-7	Retained tetra- and hexavalent actinides; Am(III) not retained from nitric solutions	Separation of Am-Pu fraction from U-Th fraction	Am and lanthanides flowed through the UTEVA resin, while Pu(IV) and U retained on UTEVA resin	[90]
DGA	N,N,N′,N′ tetraoctyldiglycolamide	Amberchrom CG-71	DGA had very strong affinity to Am; the distribution coefficient for Am was higher than 10^4^in HNO_3_(≥1M)	Separation of Pu and Am using single resin column	DGA resin could be used for quantitative separation of Am from various matrices	[91,92,93]
DIPEX	bis(2-ethylhexyl)methane diphosphonic acid	inert polymeric	DIPEX resin exhibited very strong affinity for actinides, including trivalent actinides	Pre-concentration of ^241^Am	The use of this resins was significantly limited mainly due to difficulties in recovering ^241^Am from the resin	[88]
DIPHONIX	geminally substituted diphosphonic acid ligands	styrene-based polymeric matrix	DIPHONIX resin exhibited very strong affinity for actinides	Pre-concentration of ^241^Am	The use of this resins was significantly limited mainly due to difficulties in recovering ^241^Am from the resin	[94,95]

**Table 6:** The major parameters of combined procedures for the determination of ^241^Am in environmental samples developed in recent years.

No	Sample	Pre-Treatment	Pre-Concentration	Chemical Separation	Source Preparation	Chemical Yield	Reference
	Matrix (Amount)						
1	IAEA414	16 M nitric acid digestion	Iron oxide	DGA resin	NdF_3_ microprecipitates		[92]
2	Soil sample (10 g)	Sodium hydroxide fusion	Iron hydroxide precipitate	TEVA and DGA resin, less than 8 h	CeF3 microprecipitates	89.2%	[51]
3	Liquid waste	Evaporated to dryness; acid digestion	Oxlate acid	Pu: Dowex1 × 8 resin Am: TRU resin	Electrodeposition of H_2_SO_4_-(NH_4_)_2_SO_4_	Not given	[53]
4	Low-level liquid radioactive waste	Evaporated to dryness; acid digestion	Coprecipitation on iron(II) hydroxide and calcium oxalate precipitate	Pu, Np, and U: UTEVA Am: TRU resin	NdF_3_ microprecipitates	55%	[7]
5	Soil samples (10–15 g)	Acid total dissolution with HCl, HNO_3_, HF, and HClO_4_	Leachate was filtered	Pu: AG1 × 8r resin U: UTEVA resin purification ^241^Am: TRU resin Separation of americium from lanthanides: TEVA resin	Electrodeposition	85.5%	[23]
6	Radioactive sludge from nuclear power plant	Concentrated HNO_3_		Single multi-stage column Pu: AnaLig^@^ Pu-02 resin Sr: AnaLig^@^ Sr-01 resin Am: DGA resin	NdF_3_ microprecipitates	>90%	[96]
8	Urine (25–300 mL)	Added HNO_3_	Loading after pre-filtering	Pu: AnaLig^@^ Pu-02 resin Sr: AnaLig^@^ Sr-01 resin Am: DGA resin	NdF_3_ microprecipitates	25 mL:98% 300 mL: 41%	[97]
9	Urine	Concentrated HNO_3_ and 2 M Al(NO_3_)_3_ added to adjust the acidity of each sample	Calcium phosphate precipitation	Sr: Sr resin Am: TEVA and TRU resin	CeF_3_ microprecipitates	Nearly 100%	[98]
10	Liquid waste (10 mL)	Leaching with HNO_3_		Pu: anion-exchange resin Am: TRU and anion-exchange resin	Microprecipitation and electrodeposition	77–86%	[99]
11	Soil and sediment	Leaching with HNO_3_ and HCl; total acid digestion with HNO_3_, HCl, and HF; microwave digestion with HNO_3_ and HF	Calcium oxalate precipitation	Sr: Sr-spec^@^ resin U: UTEVA resin Separation of Pu from Am: AG1X8 resin Am: mixed anion- and cation-exchange and TRU resin	Electrodeposition of H_2_SO_4_-NaHSO_4_	65–85%	[100]
12	Large-sized soil and sediment samples	Lithium metaborate fusion	Iron hydroxide precipitation and CeF_3_ coprecipitation	Pu: AGMP-1M and TEVA resin Am and Cm: DGA, AGMP-1M, and TEVA resin	CeF_3_ microprecipitation	91%	[101]
13	Large-sized soil and sediment samples	Ashing; acid digestion	Iron hydroxide precipitation, CeF_3_ coprecipitation, and fluoride coprecipitation	Pu: AGMP-1M and TEVA resin Am and Cm: DGA, AGMP-1M, UTEVA, and GDA resin	CeF_3_ microprecipitation	67.5–95.4%	[102]

**Table 7:** Overview of flow approaches developed for ^241^Am determination.

Purpose	Radionuclides	Sample Type	Flow System Design	Chemical Separation	Measurement Technique	Performance	Reference
Environmental radioactivity monitoring	^239+240^Pu and ^241^Am	Soil, vegetable ash leachate, urine, and blood	MSFIA-MPFS	Extraction chromatography (0.08 g TRU resin)	Low-background proportional counter	Chemical yield: <90% for both Pu and Am; LOD: 4 Bq/L; precision: 3%; turnover time (online separation): 40 min	[109]
	^90^Sr, ^234^U, ^241^Am, and ^239^Pu	Lake water	MSFI	Extraction chromatography (DGA-B resin)	ICP-MS	The limits of detection were 1.48 pg/L for ^90^Sr, 1.75 pg/L for ^234^U, 0.65 pg/L for ^241^Am, and 0.56 pg/L for ^239^Pu	[110]
	^237^Np, ^233^U, ^241^Am, and ^242^Pu	Artificial solution	MSFI	Extraction chromatography (UTEVA and AG-1 resins)	Alpha spectrometry	Recovery yields: 94.2% for ^233^U, 87.2% for ^237^Np, 82.1% for ^242^Pu, and 98.7% for ^241^Am	[111]
	^232^Th, ^237^Np, ^238^U, ^241^Am, and ^242^Pu	Large, spiked soil samples	Pressurized injection (PI)	Extraction chromatography (TEVA and DGA resins)	ICP-MS	Recovery yield: 97% for Th, U, Np, Pu, and Am	[105]
Nuclear waste management	^90^Sr, ^241^Am, and ^99^Tc	Aged nuclear waste	SI	Extraction chromatography (50 μL Sr resin, TRU resin, and TEVA resin)	Flow-through LSC	Chemical yields: 92 ± 2% for ^90^Sr and 99 ± 5% for ^99^Tc	[112]
	^230^ Th, ^233^U, ^239^Pu, and ^241^Am	Spiked sample solution in 2 M HNO_3_	SI	Extraction chromatography (0.63 mL TRU resin, 20–50 μm)	Flow-through LSC and alpha spectrometry	Chemical yields: up to 102 ±4% for ^241^Am, up to 101 ± 3% for ^239^Pu, up to 93 ±4% for ^233^U, and up to 88 ± 3% for ^230^Th	[113]
	^237^Np, ^238^Pu, ^239+240^Pu, and ^241^Am	Dissolved vitrified nuclear waste	SI	Extraction chromatography (0.63 mL TRU resin, 20–50 μm)	ICP-MS	U decontamination factor (for Pu determination): 3.0 × 10^5^	[114]
	^238^ Pu, ^239+240^Pu, ^241^Am, ^243+244^Cm, and ^242^Cm	Vitrified glass waste, aged irradiated nuclear fuel, and waste from Handford site	SI	Extraction chromatography (0.63 mL TRU resin, 20–50 μm)	Flow-through LSC and alpha spectrometry	Chemical yields: 85% for Pu and 86% for Am	[103]

Footnotes: flow injection (FI), sequential injection (SI), multi-commuted flow injection (MCFI), multi-syringe flow injection (MSFI), multi-pumping flow system (MPFS), and pressurized injection (PI).

**Table 8:** Isobaric and polyatomic interferences of ^241^Am in ICP-MS measurement.

Isobaric and Polyatomic Interferences	Abundance of Interference Isotopes
^241^Pu	
^240^Pu^1^H	
^209^Bi^32^S	^209^Bi: 100%
^209^BiO_2_	^209^Bi: 100%
^208^Pb^16^O_2_^1^H	^208^Pb: 52.4%
^206^Pb^35^Cl	^206^Pb: 24.1%
^204^Pb^37^Cl	^204^Pb: 1.4%
^207^Pb^34^S^+^	^207^Pb: 22.1%
^208^Pb^33^S	^208^Pb: 52.4%
^201^Hg^40^Ar	^201^Hg: 13.2%
^179^Hf^14^N^16^O_3_	^179^Hf: 13.6%
^178^Hf^14^N^16^O_3_^1^H	^178^Hf: 27.3%
^204^Hg^37^Cl	^204^Hg: 6.9%
^195^Pt^14^N^16^O_2_	^195^Pt: 33.8%
^194^Pt^14^N^16^O_2_^1^H	^194^Pt: 33.0%
^203^Tl^38^Ar	^203^Tl: 29.524%
^205^Tl^36^Ar	^205^Tl: 70.476%

**Table 9:** Comparison of LODs obtained by different ICP-MS instruments for ^241^Am measurement.

MS Techniques Used	Matrix/Separation Method or Combined Method	Limit of Detection (LOD) or Limit of Quantitation (LOQ)	Reference
Q-ICP-MS	Water and urine/TRU resin	40–150 fg/g (LOD)	[141]
Q-ICP-MS	Urine/flow injection and extraction chromatography	0.9–8 fg/g (LOD)	[142]
Q-ICP-MS	Sediment/TEVA and DGA resins	2 pg (LOD)	[143]
Q-ICP-MS, He-NH_3_ as collision–reaction gas	Soil samples IAEA-Soil-6 and IAEA-375/DGA resin	0.094 fg/g (LOD)	[26]
Q-ICP-MS, O_2_/He-He as collision–reaction gas	Soil samples IAEA-Soil-6 and IAEA-375/UTEVA and DGA resins	0.019 fg/g (LOD)	[139]
SF-ICP-MS	River sediment, human liver and lung samples/extraction with ammonium hydrogen oxalate	1.2 fg/g (LOD)	[107]
SF-ICP-MS	Sediment/TRU resin	0.9 fg/g (LOD)	[144]
SF-ICP-MS	Sediment IAEA-384, sediment IAEA-385, and seaweed IAEA-308/CaF_2_ precipitation and TRU resin	0.86 fg/g (LOD)	[145]
SF-ICP-MS	Sediment IAEA-368/isotope dilution with ^243^Am, CaF_2_ precipitation, and TRU resin	0.32 fg/g (LOD)	[138]
SF-ICP-MS	Large soil samples/Ca_2_C_2_O_4_ precipitation; TEVA and DGA-N resins	0.094 fg/g (LOD)	[137]
SF-ICP-MS	Soil and sediment/Fe(OH)_3_ precipitation; UTEVA, DAG, and TEVA resins	0.31 fg/g (LOD)	[146]
SF-ICP-MS	River water/TEVA, TRU, and Sr resins	73 fg/g (LOD)	[147]
LA-SF-ICPMS	Mosses	3.7 pg/g (LOD)	[148]
MC-ICP-MS	IAEA-385 sediment, and NIST-4350B sediment/oxalate coprecipitation; TEVA-ammonium thiocyanate column and acetone-HCl MP1 anion column	1.4 fg (LOQ)	[140]

**Table 10:** Summary of soluble species of Am in natural water.

Valence	Complex	Species
Am(III)	Hydroxide	[Am(OH)_2_]^+^
		[Am(OH)_3_]
Am(III)	Halides	[AmF_2_^+^]/[AmCl_2_^+^]
		[AmF^2+^/[AmCl^2+^]
Am(III)	Phosphates	[AmH_2_PO_4_^2+^]
Am(III)	Nitrates	[AmNO_3_]^2+^
Am(III)	Carbonates	[AmCO_3_]^+^
		[Am(CO_3_)_2_]^−^
		[Am(CO_3_)_3_]^3−^
		[AmHCO_3_]^−^
Am(III)	Silicate	[AmSiO(OH)_3_]^2+^

**Table 11:** Typical sequential extraction reagents and conditions for the fractionation of ^241^Am in soil and sediment.

Desired Geochemical Phases	Extraction Reagents	Temperature (°C)	Time (h)
1. Water soluble, exchangeable	H_2_O, MgCl_2_ 0.4M; pH 4.5	room	1
2. Carbonates	NH_4_Ac 1M, 25%Hac; pH 4	room	2
3. Oxides (Fe, Mn)	NH_2_OH.HCl 0.04M, HAc	room	5
4. Organic matter	H_2_O_2_ 30%, HNO_3_ 0.02M; pH 2	85	5
5. Residue	HNO_3_, HCl, HF, HClO_4_	100	1

**Table 12:** Distributions of ^241^Am in different fractions in sea sediment.

Location/Sample	Fractionation of ^241^Am	Reference
Venice Lagoon (northern Adriatic Sea, Italy)/VLAS	carbonates > 90%	[175]
Gaeta Gulf (central Tyrrhenia Sea, Italy)/GGTS	carbonates > 60%	[175]
Marshall Islands (central Pacific Ocean)/IAEA-367	carbonates 65%, organic matter 31%	[175]
Sellafield (Irish Sea, UK)/IAEA-135	carbonates 65%, organic matter 25%	[175]
Baltic Sea/sediment	carbonates 21%, organic matter 42%	[176]

## Data Availability

Not applicable.

## Abbreviations

AMS	Accelerator-based mass spectrometry
BTBP	2,6-bis(5,6-dialkyl-1,2,4-triazinyl-3-yl)dipyridine
BTP	2,6-bis(5,6-dialkyl-1,2,4-triazinyl-3-yl)pyridine
CA-BTP	bis-2,6-(5,6,7,8-tetrahydro-5,9,9-trimethyl-5,8-methano-1,2,4-benzotriazin-3-yl) pyridine
CMPO	octyl, phenyl-N, N-di-iso-butyl carbamoylmethyl phosphine oxide
CRM	certified reference material
CyMe_4_BTBP	6,6′-bis(5,5,8,8-tetramethyl-5,6,7,8-tetrahydrobenzo [1,2,4]triazin-3-yl)-2,2′-bipyridine
CyMe_4_BTPhen	2,9-bis(5,5,8,8-tetramethyl-5,6,7,8-tetrahydro-benzo [1,2,4]triazin-3-yl)-1,10-phenanthroline
D_Am_	weight distribution ration
DDCP	dibutyl-N,N′-diethylcarbamylphosphonate
DF	decontamination factor
DGA	di glycol amide
DIPEX	a commercial resin consisting of bis(2-ethylhexyl)methanediphosphonic acid
DIPHONIX	a commercial resin containing sulfonic and gem-diphosphonic acid groups
DTPA	2-hydorxy-1,3-diaminopropane tetra-acetic acid
EC	extraction chromatography
FI	flow injection
FPs	fission products
FWHW	full width at half maximum
HDEHP	di-2-ethylhexylphosphoric acid
IAEA	international atomic energy agency
ICP-MS	inductively coupled plasma mass spectrometry
ID	isotope dilution
LOD	limit of detection
LOQ	limit of quantification
MC	multiple-ion collector
MCFI	multi-commuted flow injection
MNPs	magnetic nanoparticles
MOB-BTP	3,3′-dimethoxy-phenyl-bis-1,2,4-triazinyl-2,6-pyridine
MPFS	multi-pumping flow system
MSFI	multi-syringe flow injection
PI	pressurized injection
PIPS	passivated ion-implanted planar silicon
PMBP	1-benzyl-3-methyl-4-benzoyl acetyl acetone
Q-ICP-MS	quadrupole ICP-MS
RE	rare-earth
SAMMS	self-assembled monolayer on mesoporous supports
SF	sector field
STS	Semipalatinsk Test Site
SI	sequential injection
TBP	tributyl phosphate
TE	total evaporation
TEVA	tetravalent actinide
TIMS	thermal ionization mass spectrometry
TIOA	tri-iso-octylamine
TOA	trioctylamine
TONA	tri-n-octylaime
TOPO	trioctyl phosphine oxide
TRU	trans-uranium
TTA	triethylene tetramine
UTEVA	uranium and tetravalent actinides
WHO	World Health Organization
XRD	X-ray diffraction
